# Endocrine disruption and male reproductive disorders: unanswered questions

**DOI:** 10.1093/humrep/deae143

**Published:** 2024-06-26

**Authors:** Richard M Sharpe

**Affiliations:** Centre for Reproductive Health, Institute for Regeneration & Repair, The University of Edinburgh, Edinburgh, UK

**Keywords:** endocrine-disrupting chemicals, EDCs, phthalates, Western diet, androgens, sperm count, masculinization programming window, foetal growth impairment, medications

## Abstract

Maternal exposure to endocrine-disrupting chemicals (EDCs) in human pregnancy is widely considered as an important cause of adverse changes in male reproductive health due to impaired foetal androgen production/action. However, the epidemiological evidence supporting this view is equivocal, except for certain phthalates, notably diethyl hexyl phthalate (DEHP). Maternal phthalate exposure levels associated with adverse reproductive changes in epidemiological studies are several thousand-fold lower than those needed to suppress foetal androgen production in rats, and direct studies using human foetal testis tissue show no effect of high phthalate exposure on androgen production. This conundrum is unexplained and raises fundamental questions. Human DEHP exposure is predominantly via food with highest exposure associated with consumption of a Western style (unhealthy) diet. This diet is also associated with increased exposure to the most common EDCs, whether persistent (chlorinated or fluorinated chemicals) or non-persistent (phthalates, bisphenols) compounds, which are found at highest levels in fatty and processed foods. Consequently, epidemiological studies associating EDC exposure and male reproductive health disorders are confounded by potential dietary effects, and vice versa. A Western diet/lifestyle in young adulthood is also associated with low sperm counts. Disentangling EDC and dietary effects in epidemiological studies is challenging. In pregnancy, a Western diet, EDC exposure, and maternal living in proximity to industrial sites are all associated with impaired foetal growth/development due to placental dysfunction, which predisposes to congenital male reproductive disorders (cryptorchidism, hypospadias). While the latter are considered to reflect impaired foetal androgen production, effects resulting from foetal growth impairment (FGI) are likely indirect. As FGI has numerous life-long health consequences, and is affected by maternal lifestyle, research into the origins of male reproductive disorders should take more account of this. Additionally, potential effects on foetal growth/foetal testis from the increasing use of medications in pregnancy deserves more research attention.

## Graphical Abstract

**Graphical Abstract:**
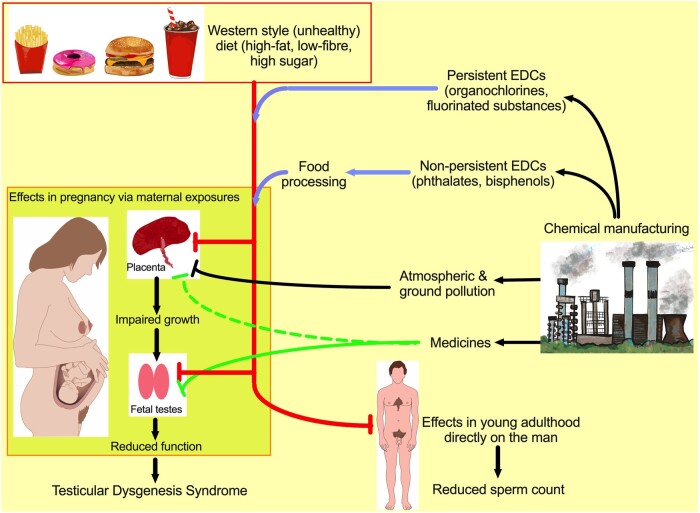
**Pathways via which diet and exposure to endocrine-disrupting chemicals, medicines, and industrial pollution can inter-connect to affect reproductive development/health of men.** EDCs, endocrine-disrupting chemicals.

## Introduction

It is widely accepted that human male reproductive health has changed for the worse over the past 75 years. This is evidenced by a progressive increase in risk of testicular germ cell cancer (TGCC), a probable increase in risk of cryptorchidism and hypospadias, and a decrease in sperm count among young men—collectively, this has been termed the testicular dysgenesis syndrome (TDS: Skakkebaek *et al.*, 2016; Levine *et al.*, 2017, 2023). The fact that these changes have occurred over a relatively short time period points to an environmental/lifestyle cause(s) (Skakkebaek *et al.*, 2016). In this regard, the widely accepted prime suspect is increased *in utero* exposure of the male foetus to pollutant/contaminant endocrine-disrupting chemicals (EDCs), notably certain phthalates, pesticides, bisphenol A, polyfluorinated compounds etc (references below). More recently, exposure to painkiller medications has been added to the exposure list (Kilcoyne and Mitchell, 2019). It is around 30 years since EDCs thrust their way into the limelight and became established as the prime suspect cause of male reproductive health disorders (Toppari *et al.*, 1996). Despite this lengthy timescale, the evidence implicating EDCs as causal agents for TDS in human males is far from conclusive. While there are claims that the indirect evidence is sufficient to merit a conviction (e.g. Hauser *et al.*, 2015), when the evidence is dissected, inconsistencies and confounders emerge. Arguably, the most important questions in this area are unanswered, ignored, or at best not answered unequivocally; this is the focus of this review.

**Figure 1.**
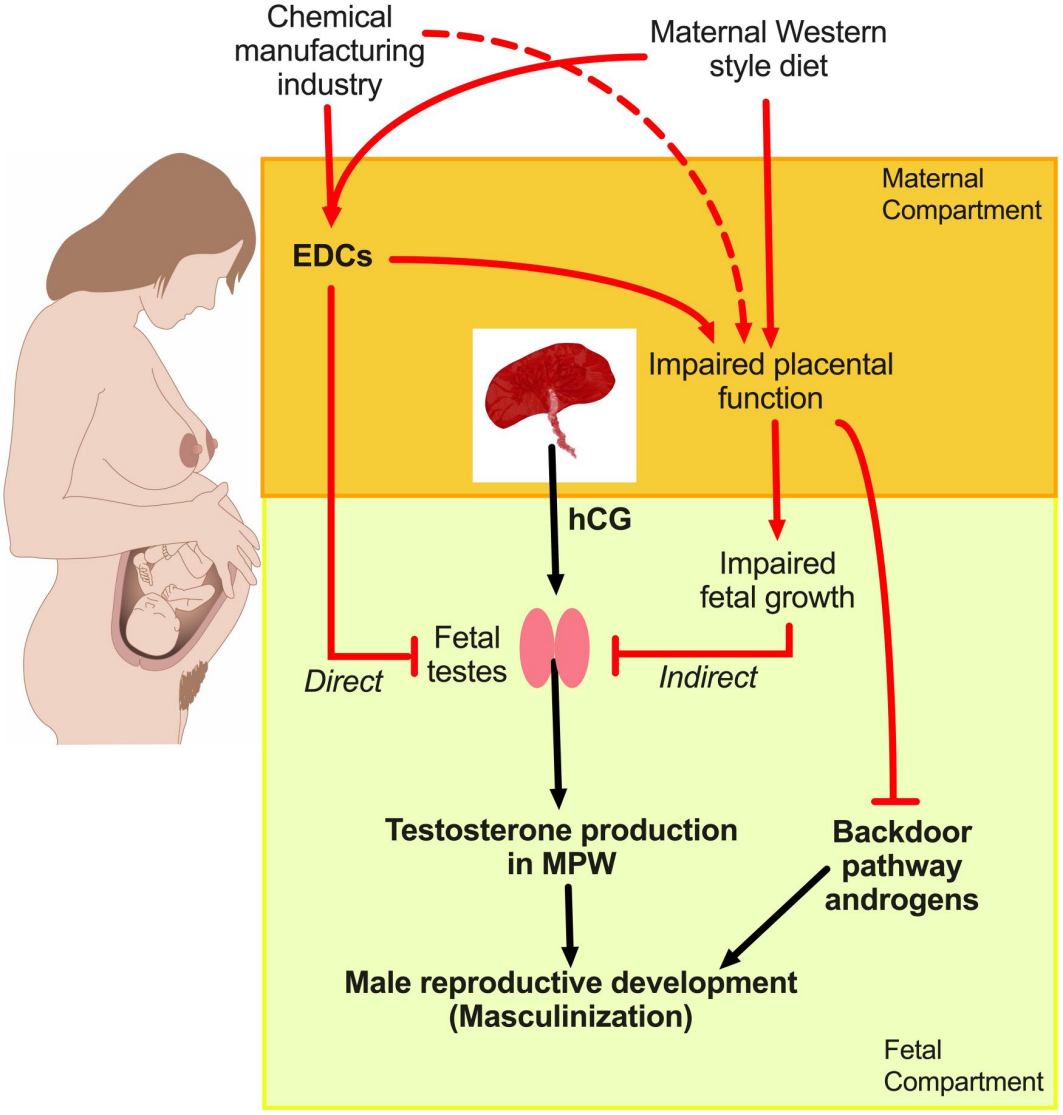
**Pathways via which maternal diet and/or exposure to endocrine-disrupting chemicals or direct exposure to industrial waste/emissions might impair androgen-dependent masculinization of the male foetus.** The normal masculinization pathway is shown by black lines and the potential direct and indirect pathways of impairment of this process are shown by red lines. EDCs, endocrine-disrupting chemicals; MPW, masculinization programming window.

Before detailing these questions, it is important to emphasize the key role that ‘TDS research’ has played in revealing that reproductive health among young men has declined within the past century (Skakkebaek *et al.*, 2016). This has occurred in an era when couples in developed countries are delaying trying for a family until the female partner is in her 30s, when her fertility is in progressive decline (Broekmans *et al.*, 2009). Consequently, this has placed additional demands on the fertility potential of the male partner (Sharpe, 2012; Skakkebaek *et al.*, 2022). Low sperm counts in many young men (Levine *et al.*, 2023) mean that the couple will take longer to achieve a pregnancy, when time is not on their side, raising the prospect of increasing couple fertility problems at a time when couple fecundity is below population replacement levels (Sharpe, 2012; Skakkebaek *et al.*, 2022); this situation is made worse by a Western-style diet in the female partner (e.g. Grieger *et al.*, 2018). From the mechanistic perspective, clinical studies and experimental studies in animals have worked in tandem to formulate new concepts and hypotheses, which have begun to identify how and when male reproductive health disorders might originate in a ‘masculinization programming window’ (MPW) in early foetal life (Sharpe, 2020; Fig. 1). In turn, this has opened up new clinical research possibilities by identifying measurable endpoints that can inform on otherwise hidden foetal events in the first trimester of pregnancy, as outlined below.

The evidence for EDC involvement in human TDS disorders comes from animal experimental studies and human epidemiological studies.

## EDC exposure and TDS disorders in experimental animals

Studies in rats have shown that foetal exposure to certain phthalates (e.g. diethyl hexyl phthalate: DEHP, or dibutyl phthalate: DBP) or to pesticides/fungicides with intrinsic anti-androgenic activity can induce TDS-like disorders in resulting male offspring, primarily cryptorchidism and hypospadias (Furr *et al.*, 2014; Schwartz *et al.*, 2019). The most detailed studies have involved exposure of pregnant rats to DEHP or DBP and have revealed that only exposure in a discrete time window (embryonic days 15.5-18.5) causes increased incidence of TDS disorders in male offspring (van den Driesche *et al.*, 2017). This key foetal time window has been termed the MPW (Welsh *et al.*, 2008; van den Driesche *et al.*, 2017). Numerous studies have shown that DBP or DEHP exposure in the MPW also results in a reduction in anogenital distance (AGD) in exposed males that is evident at birth and persists for life (e.g. van den Driesche *et al.*, 2017; Schwartz *et al.*, 2019). In normal male rats, AGD is ∼2-fold longer than in normal females, a difference that is solely attributable to exposure to androgens from the foetal testis during the MPW (Sharpe, 2020). Remarkably, equivalent DBP exposure of rats during the foetal period (e19.5–e21.5) immediately after the MPW does not reduce AGD or induce TDS disorders despite causing an equal reduction in foetal testis androgen production (van den Driesche *et al.*, 2017).

## EDC exposure and TDS disorders in humans

The importance of the robust association between foetal androgen exposure in the MPW and resulting AGD in rat studies is that it has enabled translation of experimental studies in rats to clinical observational studies in humans, in whom a similar male–female difference in AGD is evident (reviewed in Sharpe, 2020). Thus, AGD measurement at birth or in later life provides an indirect means of retrospectively ‘measuring’ foetal androgen exposure in the presumptive MPW and relating this to exposures of the mother during pregnancy. The available evidence places the MPW in humans within the period 8–14 weeks’ gestation (Welsh *et al.*, 2008; Sharpe, 2020). Various studies in human males have shown that reduced AGD (or the alternative measure anoscrotal distance) is associated with TDS disorders, although the associations are not as robust as in rat experimental studies (Dean and Sharpe, 2013; Schwartz *et al.*, 2019; Sharpe, 2020). In this regard, not all cases of an individual TDS disorder (e.g. cryptorchidism) may present with a subnormal AGD (e.g. Cortes *et al.*, 2024), which might indicate that not all cases originate because of reduced androgen exposure in the MPW. Additionally, associations between maternal EDC exposure and a TDS disorder, such as cryptorchidism, may be found but not associated with any significant reduction in AGD (e.g. Fisher *et al.*, 2020).

Numerous epidemiological studies have been undertaken to explore whether there is an association between increased maternal exposure to specific EDCs during pregnancy and consequent AGD in resulting sons and/or the occurrence of cryptorchidism or hypospadias in early postnatal life. A smaller number of studies have explored associations between AGD and adult reproductive function (sperm count, reproductive hormones). Most studies have focussed on exposure to phthalates, but others have explored exposure to persistent organochlorine compounds (e.g. dichloro-diphenyl-trichloroethane; DDT, polychlorinated biphenyls: PCBs), certain pesticides (e.g. linuron, prochloraz) and per- or poly-fluoroalkyl substances or bisphenol A-related compounds. These studies are too numerous and complex to detail here, but readers can find detailed reviews and meta-analyses in the literature (Minguez-Alarcon *et al.*, 2016; Dorman *et al.*, 2018; Sun *et al.*, 2018; Zarean *et al.*, 2019; Nelson *et al.*, 2020). The take-home message from these reviews is that, in general, the relationship between maternal exposure to the compound(s) in question and reduced AGD in sons is inconsistent or absent. The exception concerns exposure to certain phthalates, in particular DEHP, as meta-analysis has shown that, overall, higher maternal exposure is associated with a small but significant reduction in AGD in sons around birth (Dorman *et al.*, 2018; Zarean *et al.*, 2019), although not every individual study found such a relationship (Nelson *et al.*, 2020). Moreover, the significant meta-analysis result creates a conundrum that poses several fundamental unanswered questions, which in turn has wider implications for other EDCs, as discussed below.

## Maternal phthalate exposure and reduced AGD in sons—the elephant in the room

The most remarkable aspect about the association between higher maternal exposure to DEHP and related phthalates (e.g. DBP) and reduced AGD in sons in epidemiological studies, is that it occurs at levels of phthalate exposure that are several thousand-fold *lower* than the levels required to induce such an effect in rats, which is due to phthalate-induced suppression of testosterone production by the foetal testis in the MPW (reviewed in Sharpe, 2020). However, when the sensitivity of foetal rat and human testis tissue to phthalates is compared under controlled experimental conditions in culture or after xenotranplantation into nude mice, the results are 100% consistent in showing no evidence for *any* phthalate-induced suppression of testosterone production by the foetal human testis at the high levels of phthalate exposure which cause pronounced suppression of testosterone production by the foetal rat testis (reviewed in Kilcoyne and Mitchell, 2019; Sharpe, 2020). So, in contrast to what the epidemiological studies suggest, the direct studies using human foetal testis tissue under experimental conditions show that it is not several thousand-fold more sensitive than the rat to adverse phthalate effects, but in fact the converse. Remarkably, this conundrum is only discussed sketchily, if at all, in the relevant epidemiological studies—it truly is the elephant in the room.

Two other pieces of evidence support the view that higher exposure to relevant phthalates in pregnancy is without effect on male reproductive development in humans and in a non-human primate. First, women who have been exposed to DBP levels 50- or more fold higher than in epidemiological studies, through taking medication for inflammatory bowel disease (in which DBP was used in the enteric-resistant tablet coating; Hernandez-Diaz *et al.*, 2009), did not give birth to sons with any increased incidence of congenital reproductive abnormalities (e.g. Rahimi *et al.*, 2008; Ban *et al.*, 2014; Kallen, 2014). Second, experimental exposure of pregnant non-human primates (marmosets) to phthalate levels that cause pronounced increase in congenital reproductive abnormalities in rats, did not cause any such defects in resulting male offspring (McKinnell *et al.*, 2009).

The appropriate scientific response to the ‘phthalate exposure conundrum’ outlined above is to search for evidence that would allow reconciliation of the conflicting data. A starting point is to ask how the general population get exposed to DEHP/DBP and other relevant phthalates? The answer, especially for DEHP, is that exposure is predominantly via food and drink (Rudel *et al.*, 2011; Koch *et al.*, 2013; Pacyga *et al.*, 2019; Giuliani *et al.*, 2020; Fig. 1). DEHP is highly fat- and ethanol-soluble and, accordingly, fatty foods (e.g. dairy products, vegetable oils) and alcoholic drinks usually show higher phthalate contamination than other foods and beverages (Giuliani *et al.*, 2020). DEHP enters food during preparation and processing, for example via migration from polyvinyl chloride (PVC) piping which is widely used during liquid processing, or from PVC-containing gloves or food containers used by food operatives (reviewed in Giuliani *et al.*, 2020). In general, the phthalate content of food increases as it proceeds along the food preparation/processing chain (Giuliani *et al.*, 2020), and the more highly processed that foods are, the higher is their phthalate content (Rudel *et al.*, 2011; Zola *et al.*, 2016; Buckley and Kim, 2019; Pacyga *et al.*, 2019; Giuliani *et al.*, 2020). Correspondingly, phthalate exposure is notably high in ‘fast-food’ (Edwards *et al.*, 2022) or in ‘dining out’ food compared to similar fresh food prepared and eaten at home (Varshavsky *et al.*, 2018). Conversely, a ‘healthy’ diet, high in fruit and vegetables, is generally associated with lower phthalate exposure (Serrano *et al.*, 2014; Correia-Sa *et al.*, 2018; Pacyga *et al.*, 2019). As phthalate (particularly DEHP) exposure is higher if an individual is eating a less healthy, Western style, diet, it could be argued that epidemiological studies linking maternal DEHP exposure to reduced AGD in sons are actually linking an unhealthy maternal diet and AGD. Considering the numerous well-established adverse non-reproductive health effects of a Western-style diet, this is not a controversial hypothesis.

One study, in a Spanish cohort of 476 pregnant women and their resulting offspring, showed that maternal consumption of a high-fat diet was associated with a significant decrease in AGD in resulting newborn sons (Papadopoulou *et al.*, 2014). However, consumption of this diet also led to higher maternal exposure to persistent organic pollutants (POPs), and the authors suggested that it was likely the increased POP exposure that caused the reduction in AGD in sons. Unfortunately, phthalate exposure was not measured, although based on the various studies discussed above it would seem likely that mothers eating a higher fat diet would have been more highly exposed to phthalates than those eating a lower fat diet. Epidemiological studies on their own can never prove cause and effect and, as the study by Papadopoulou *et al.* (2014) illustrates, distinguishing whether it is diet *per se* or specific factors in the diet, such as contaminant EDCs, that is responsible for the reduced AGD in sons is impossible.

While the foregoing analysis is specific for phthalates such as DEHP, it appears that, in general, similar considerations may apply to other widely studied EDCs. Thus diet (especially a Western-style diet) is the major factor determining human EDC exposure in pregnancy, whether this be POPs such as PCBs, dioxins, and chlorinated pesticides (Gasull *et al.*, 2011; Marks *et al.*, 2021), poly-fluoroalkyl substances (PFAs) (Carnero *et al.*, 2021) or non-persistent compounds such as bisphenol A (Rudel *et al.*, 2011; Christensen *et al.*, 2012; Pacyga *et al.*, 2019; Russo *et al.*, 2019) and phthalates (Rudel *et al.*, 2011; Pacyga *et al.*, 2019; Giuliani *et al.*, 2020; Fig. 1). At the very least, the relationship between diet and EDC exposure highlights that in any epidemiological study of EDC exposure and specific health outcomes, diet is a huge confounding factor that is difficult to disentangle. This fundamentally impacts interpretation of any results but is rarely discussed in EDC epidemiological studies. Whether an underlying effect of maternal diet (e.g. Papadopoulou *et al.*, 2014) might explain the observed associations between maternal phthalate exposure and reduced AGD in sons (reviewed in Dorman *et al.*, 2018; Zarean *et al.*, 2019) is impossible to conclude, but if this was the case it would probably resolve the conundrum discussed above. Nevertheless, it must also be kept in mind that the Western dietary changes discussed above are clearly associated with increased exposure to a range of EDCs, which thus increases the possibility of ‘EDC mixture effects’ (Gaudriault *et al.*, 2017), but which are difficult to assess definitively in epidemiological studies involving maternal exposure.

## Medications and male reproductive health disorders

Until recently, research into the potential causes of TDS disorders has been focussed almost entirely on environmental contaminants/pollutants while ignoring pharmaceuticals, with the exception of diethylstilbestrol (Toppari *et al.*, 1996). Pharmaceuticals are designed to be strongly bioactive and human exposure is intentional and at high levels, whereas contaminants/pollutants are only weakly endocrine-active and human exposure is low and unintentional. This contrast is all the more remarkable when considering that the period in which TDS disorders have increased in incidence is a period when the number of available medications and their usage has expanded enormously, whereas contaminant levels have generally declined due to tighter regulatory controls. Most relevant to TDS origins is that nearly all pregnant women in developed countries now use one or more prescribed or over-the-counter medications, whether this be for chronic disorders (e.g. inflammatory bowel disease, asthma, epilepsy) or for short-term pain relief or temperature or infection control (Lupattelli *et al.*, 2014; reviewed in Stock and Norman, 2019). Pregnant women are prescribed an average of 2.6 medicines in the USA and 4.6 in Italy (Stock and Norman, 2019), yet as these authors and others (Parisi *et al.*, 2011) emphasize, most of the medications prescribed/used in pregnancy have not been specifically evaluated for safety in human pregnancy, this especially being the case for older medicines. An illustration of the latter is the commonest painkiller paracetamol (acetaminophen).

Up to 70% of women will take paracetamol during their pregnancy and, in the UK and elsewhere, it is recommended as safe for such use (Bauer *et al.*, 2021). However, there is growing evidence that its use in pregnancy may pose risks for the developing foetus (Zafeiri *et al.*, 2022). Most pertinent to the present review is that paracetamol use in the first trimester has been associated with shortened AGD in exposed sons at birth, although this was found in only two of three studies (reviewed in Tadokoro-Cuccaro *et al.*, 2022). This association is supported by demonstration that paracetamol, at human therapeutic exposure levels, can significantly suppress testosterone production by human foetal testis tissue (reviewed in Kilcoyne and Mitchell, 2019). Similar effects have been shown experimentally in rats (Kilcoyne and Mitchell, 2019) and other *in vitro* evidence points to potential effects of paracetamol on human foetal germ cells (Hurtado-Gonzalez *et al.*, 2018). However, the key question is whether maternal paracetamol use during early pregnancy is for long enough to suppress foetal testosterone production sufficiently to result in endpoint effects, such as cryptorchidism or hypospadias, in sons. The available data, which includes meta-analysis of 10 studies, suggests that there is only weak (inconsistent) evidence for an association between maternal paracetamol use and cryptorchidism in sons and little evidence for an association with hypospadias (reviewed in Gurney *et al.*, 2017; Tadokoro-Cuccaro *et al.*, 2022). Furthermore, as maternal paracetamol use is associated with increased risk of several perinatal outcomes, including reduced birthweight and increased prematurity (Zafeiri *et al.*, 2022), which are themselves key risk factors for cryptorchidism and hypospadias (Fujimoto *et al.*, 2008; Jensen *et al.*, 2012), the weak associations found in epidemiological studies may be due to this confounding rather than to direct impairment of testosterone production by the foetal testis (Fig. 1).

While the reviews cited above suggest that paracetamol use alone may not pose a huge risk to human male reproductive development in foetal life, it does ask whether any other medicines used by women in pregnancy might have adverse effects on the foetal testis. One study of a Danish birth cohort found that maternal use of azole antifungal medicines, which is common in pregnancy, was associated with shortened AGD in sons (Mogensen *et al.*, 2017). A retrospective cohort study in Australia of >12 000 women who underwent assisted reproduction, found that in those (n = 618) who were administered corticosteroid as part of the treatment process there was a >3.7-fold increase in risk of cryptorchidism or hypospadias in resulting sons, when compared with women who did not receive corticosteroid during their assisted reproduction (Thalluri *et al.*, 2022). Lastly, Tavlo *et al.* (2022) have suggested that the widespread use of metformin during pregnancy might pose risks to foetal male development, based on studies in mice and using human foetal testis tissue, which show metformin inhibition of testosterone production (Tartarin *et al.*, 2012). However, a small randomized trial that compared metformin versus insulin for the treatment of gestational diabetes (Tertti *et al.*, 2016) found no effect of metformin on resulting infant testis size, although testosterone levels were not measured and treatment did not commence until 2 months after the end of the presumptive MPW.

## Adult onset TDS disorders in young men—is it solely down to effects within the MPW?

The foetal origin of cryptorchidism and hypospadias is self-evident and the case for this and associated changes in AGD resulting from disruption of androgen production/action within the MPW is supported by several pieces of direct evidence (reviewed in Schwartz *et al.*, 2019; Sharpe, 2020). However, it is not clear how such ‘androgenic effects’ within the MPW could account for occurrence of TGCC or low sperm count in adulthood.

Germ cells are not direct androgen targets at any stage in life (O’Hara and Smith, 2015) and although Sertoli cells, which guide and support germ cell development throughout life, are an important androgen target essential for normal spermatogenesis (O’Hara and Smith, 2015), they do not express the androgen receptor (AR) during foetal and early neonatal life when testicular testosterone levels are elevated (Sharpe *et al.*, 2003; Chemes *et al.*, 2008; O’Hara and Smith, 2015). However, as the AR is expressed in the foetal testis by peritubular myoid cells (O’Hara and Smith, 2015) and by non-Leydig interstitial cells (thought to be progenitor cells for adult Leydig cells; Kilcoyne *et al.*, 2014), altered function of these cells could potentially mediate androgen deprivation effects on Sertoli and/or germ cells, although the pathways involved are unknown.

TGCC is established to originate in perinatal life due to failure of some foetal germ cells to differentiate normally into pre-spermatogonia (Rajpert-De Meyts, 2006). Moreover, demonstration that men with TGCC exhibit a shorter AGD than tumour-free normozoospemic controls (Moreno-Mendoza *et al.*, 2020) or tumour-free control men from the general population (Priskorn *et al.*, 2021) provides a link to altered androgen action in the MPW. Similarly, smaller adult testis size and lower sperm count have also been associated with shorter AGD in adult men, although these associations are not as robust as those for cryptorchidism and hypospadias (Dean and Sharpe, 2013; Sharpe, 2020). As the main determinant of sperm production/sperm count in adulthood is the number of Sertoli cells, and these cells proliferate in foetal, neonatal, and peripubertal life in man (Sharpe *et al.*, 2003), it is unclear why any adverse effects in foetal life on Sertoli cell proliferation/number would not be compensated for by increased proliferation after birth (Sharpe *et al.*, 2003). One suggestion, based on observational data, is that adverse foetal events might impair the ability of a proportion of Sertoli cells to undergo maturation during puberty (Sharpe *et al.*, 2003), as this would render them incapable of supporting normal spermatogenesis but might enable continued support of foetal germ cells, hence TGCC (see Sharpe and Mitchell, 2013). Another interpretation of the data is that TDS disorders result because of an overall abnormality in foetal testis development (e.g. due to foetal growth impairment; Fig. 1). This being the case, the association of shortened AGD with all TDS disorders would not necessarily indicate that the disorder in question resulted *per se* from a direct effect on the pathways of androgen production/action, simply that impaired foetal testis development resulted in abnormal development and function of all of the testicular cell types, whether Leydig, Sertoli, or germ cells (Sharpe and Skakkebaek, 2008; Skakkebaek *et al.*, 2022). From the causal perspective, this would mean that research attention should not be restricted to exposures that directly cause foetal testis endocrine dysfunction but rather to exposures that impair overall growth and development of the foetal testis, such as those that affect placental development and function, as discussed below.

## Low sperm counts—foetal effects versus postnatal effects, and EDCs versus diet

Several mother-son cohort studies have assessed whether maternal exposure to certain EDCs is associated with sperm count/semen quality of sons in adulthood. One study (n = 101) found no relationship between maternal bisphenol A exposure and either AGD at birth or semen quality of sons in adulthood (Holmboe *et al.*, 2022). A similar size study found no significant relationship between maternal phthalate exposure and semen quality of sons (Henriksen *et al.*, 2023). Another study of 184 men found only marginal evidence for a negative association between airborne PCB exposure during foetal life and testis size and semen quality in adulthood (Tøttenborg *et al.*, 2022). Conversely, a cohort study of 864 young men did show a consistent association between maternal exposure to per-fluoroalkyl substances and PFAs in early pregnancy and sperm count (but not testis size) in sons in adulthood (Hærvig *et al.*, 2022). Such cohort studies are incredibly demanding and expensive and, in view of the 20+ year gap between exposure (of the mother) and outcome (in the sons), obtaining high-quality definitive evidence one way or the other is challenging. Moreover, with EDCs such as those investigated in these four cohort studies, there is the ever-present confounding factor of maternal diet, as discussed earlier.

Lifestyle/environmental factors during postnatal life could also impact testis development and/or function in addition to, or instead of, impacts during foetal life. A range of studies have assessed if the diet of young men is associated with sperm count/semen quality. These have shown that adherence to a healthy, Mediterranean-style diet is associated with higher sperm counts and a modern Western-style diet is associated with lower sperm counts (Jurewicz *et al.*, 2016; Cutillas-Tolin *et al.*, 2019; Nassan *et al.*, 2020). More specifically, high intake of processed red meat (Afeiche *et al.*, 2014) or saturated fat (Jensen *et al.*, 2013) or sugar-sweetened drinks (Nassan *et al.*, 2021) is associated with a lower sperm count whereas a diet rich in fruit and vegetables is associated with a higher sperm count (Chiu *et al.*, 2020). Equally relevant to a modern Western lifestyle, caffeine intake via cola drinks (Jensen *et al.*, 2010) and e-cigarette use (Holmboe *et al.*, 2020) are both associated with lower sperm counts as also is a lifestyle involving sitting for >5 hours per day watching television (Priskorn *et al.*, 2016). While it is difficult to be convinced that each of the individual dietary/lifestyle factors cited is negatively impacting sperm counts, viewed overall it is hard to escape the conclusion that the modern diet and lifestyle of young men in the West is having a negative effect on their sperm counts, irrespective of whether or not their sperm count has been affected earlier by events *in utero*. It should also be kept in mind that the dietary factors associated with lower sperm counts in young men are also likely to increase exposure to several EDCs, as discussed earlier for pregnant women.

## Factors, including EDCs, affecting placental function, prematurity, and birthweight

The most important factor identified as increasing risk of cryptorchidism and hypospadias is low birthweight (Jensen *et al.*, 2013), especially for hypospadias when this stems from placental dysfunction in the first trimester (e.g. Fujimoto *et al.*, 2008; Toufaily *et al.*, 2018; Fig. 1). There is abundant evidence that residential proximity of women in pregnancy to industrial sites, especially hazardous waste sites and landfills, is associated with increased risk of prematurity, low birthweight, and small for gestational age (e.g. Kihal-Talantikite *et al.*, 2017; Bergstra *et al.*, 2021) as well as increased risk of cryptorchidism and/or hypospadias (Kihal-Talantikite *et al.*, 2017; Le Moal *et al.*, 2021). Increased exposure to air pollution in such areas has also been associated consistently with similar increased perinatal risks (e.g. Porter *et al.*, 2014; Bergstra *et al.*, 2021).

More pertinent to this review, increased exposure to persistent organochlorine compounds/pesticides as well as to heavy metals (reviewed in Ferguson and Chin, 2017) or PFAs (Waterfield *et al.*, 2020) or to the phthalate DEHP (Ferguson *et al.*, 2014) have all been associated with increased risk of preterm birth (Fig. 1), and in the case of DEHP this was shown to be ‘dose-related’. As detailed earlier, increased exposure to these various EDCs is also associated with adherence to an unhealthy ‘Western-style’ diet (high saturated fat, high sugar, high processed food intake), and such a diet in pregnancy is itself associated with lower birthweight, based on a detailed systematic review (Chia *et al.*, 2019; Fig. 1). Conversely, the same review showed that maternal adherence to a healthy dietary pattern is associated with decreased risk of preterm birth and higher birthweight, and would likely also reduce exposure to the compounds discussed above. Consequently, if diet and/or associated chemical exposures play a causal role in the aetiology of TDS disorders as a consequence of effects on placental function and foetal growth (Fig. 1), disentangling the potential effect of EDCs from that of diet in this regard appears as intractable as discussed earlier for associations with AGD changes in sons.

Another important consequence of any impact of diet and/or EDC or other chemical exposures (whether diet-related or not) on placental development/function is that they may negatively impact foetal androgen exposure during the MPW in two ways. First, general delay of foetal growth may result in parallel delay of foetal testis development/function with a consequent reduction in testosterone production (Fig. 1). It is notable that hCG produced by the placenta is both the driver of testosterone production by the human foetal testis during the MPW (see Scott *et al.*, 2009; Sharpe, 2020; Adibi *et al.*, 2024) and an important driver of early placental development and foetal growth (Adibi *et al.*, 2024). Second, in human male reproductive development, normal masculinization requires not only testosterone from the foetal testis but also androgens produced via the so-called backdoor pathway (Fig. 1) in which normal placental function plays a key role, independent of hCG production (for details see O’Shaughnessy *et al.*, 2019; Sharpe, 2020).

## Conclusions and future research approaches

Based on current understanding of the importance of the MPW in normal male reproductive development, it is understandable that research into the causes of TDS disorders has focussed on maternal exposure to EDCs that might disrupt normal foetal testis endocrine function. However, as this review has hopefully shown, the evidence that individual EDCs negatively impact human male reproductive development is either inconsistent or heavily confounded by dietary changes and choices. This is not to say that EDCs do not have some impact, especially when considering exposure to EDC mixtures (which is more akin to the ‘real world’; Gaudriault *et al.*, 2017) and/or indirect effects on the foetal testis mediated by overarching placental/foetal growth effects. When pointing out the extent to which human EDC exposure studies are subject to confounding, this is not to dismiss the potential for EDC effects but rather to emphasize that any such effects are difficult to disentangle from potential effects of diet—and conversely, the same confounding applies to potential effects of diet seen in epidemiological studies, as these are potentially confounded by exposure to a range of EDCs.

Irrespective of these confounding issues, it is possible to conclude unequivocally that man-made changes in pollution/contamination as a result of industrialization, and man-made changes in our diets (i.e. Western-style diet), and inadvertent chemical exposures resulting from such dietary changes, are clearly associated with adverse male reproductive changes, whether due to exposures via the mother in foetal life or directly to the male in postnatal life. The fact that the reproductive, dietary, and EDC exposure changes discussed in this review have all occurred over a similar timescale reinforces this conclusion. Based on the available evidence, it is impossible to be certain whether it is diet or resulting chemical exposures (or both) that might be responsible for human male reproductive changes. It is also not possible to conclude whether any effects of maternal diet/EDC exposure on male reproductive health are the result of direct perturbation of foetal Leydig cell testosterone production (i.e. endocrine disruption *per se*) or are a consequence of overall disruption of foetal testis development, which leads secondarily to reduced testosterone production.

In terms of future research priorities, there is no easy path towards disentangling dietary and EDC exposure associations in epidemiological studies. It is therefore perhaps sensible to focus more on studies to dissect the role that placental function plays in foetal growth, and thus foetal testis development, bearing in mind the evidence that links impaired foetal growth to maternal diet (Chia *et al.*, 2019), lifestyle (Reijnders *et al.*, 2019), EDC exposures (Ferguson *et al.*, 2014; Ferguson and Chin, 2017; Waterfield *et al.*, 2020), and place of residence (e.g. Kihal-Talantikite *et al.*, 2017; Bergstra *et al.*, 2021; Le Moal *et al.*, 2021). As foetal growth is critically determined by placental development and function, including production of the molecules (hCG, progesterone) that drive foetal androgen exposure in human males (Sharpe, 2020), new insights into the underlying (foetal) causes of male reproductive maldevelopment will likely emerge. Despite the unknowns and uncertainties detailed above, it is possible to make one very strong and unequivocal recommendation based on all of the available evidence, namely that adopting a healthier, Mediterranean-style diet in pregnancy and in young male adulthood will not only improve male reproductive health and general health, but also will reduce exposure to a range of EDCs and other environmental chemicals; a rare win-win situation!

## Data Availability

The data and manuscripts cited in this review are all from the peer-reviewed literature and are readily accessible via PubMed or Google Scholar or via institutional access.
